# Viscosupplementation may preserve tibial cartilage and collagen in osteoarthritis: findings from a preclinical model of osteoarthritis

**DOI:** 10.1186/s40634-020-00256-4

**Published:** 2020-05-31

**Authors:** John Lokhnauth, Kevin E. Driscoll, Alison Bendele, Faizan Niazi, Alfred Liang, Crilles C. Larsen

**Affiliations:** 1 Ferring Pharmaceuticals Inc., 100 Interpace Parkway, Parsippany, NJ 07054 USA; 2 Healthcare Innovation Partners, Mountain Lakes, NJ USA; 3 Bolder BioPATH, Boulder, CO USA

**Keywords:** Osteoarthritis, Hyaluronic acid, Collagen, Viscosupplementation

## Abstract

**Purpose:**

Intraarticular (IA) hyaluronic acid (HA) injection is used to reduce pain and improve mobility in knee osteoarthritis (OA). Little is known about histopathological changes underlying HA efficacy. This study investigated dose-related effects of 1% sodium hyaluronate (BioHA) on knee joint histopathology and pain responses in a medial meniscal tear (MMT) rat model of OA.

**Methods:**

Following MMT surgery, rats were randomized into treatment groups: single IA injection of vehicle, BioHA, or an avian-derived hyaluronic acid (hylan G-F 20) on Day 7; or 3 weekly injections of vehicle or BioHA on Days 7, 14, and 21. On Day 35, joints were evaluated by microscopic histopathology for cartilage degeneration, collagen degeneration, synovitis, and cytokine expression (tumor necrosis factor α, transforming growth factor β).

**Results:**

Joint pathology for control animals was consistent with that expected for the MMT model. Rats treated with 3 injections of IA-BioHA had significantly reduced collagen degeneration (21%) relative to control animals. No significant change in collagen degeneration was observed for rats given a single injection of hylan G-F 20 or IA-BioHA compared to control animals. HA treatment did not affect cytokine expression.

**Conclusions:**

IA-BioHA viscosupplementation in a rat MMT model of OA showed preservation of joint cartilage and collagen. This effect was most pronounced on tibial surfaces having less severe injury, suggesting that treatment should be initiated early in the disease process. A comparison of responses to IA-BioHA or hylan G-F 20 in the MMT rat OA model suggest IA-BioHA may be more effective in preserving joint connective tissue.

## Background

Osteoarthritis (OA) is characterized by synovitis, breakdown of articular cartilage, and changes in the quantity and quality of synovial fluid [16, 18]. Hyaluronic acid (HA) is a constituent of cartilage and synovial fluid that maintains articular health through its viscoelastic and lubricating properties, as well as participating in cell proliferation, migration, and hydrodynamics [2, 9]. The concentration and distribution of molecular weights of synovial HA are reported to decrease in OA [2, 20]. Viscosupplementation of synovial joints by intra-articular (IA) injection of HA has been shown to reduce pain and improve mobility in patients with symptomatic knee OA [21, 22].

Commercially-available HA products for intra-articular injection vary greatly in terms of source of HA, structure, molecular weight, concentration, volume per injection, and number of injections per course of therapy [1, 10, 13, 17]. Clinical studies have suggested that differences in HA formulations are associated with variation in their efficacy and safety performance [1, 10, 13, 17]. For example, a head-to-head, double-blind, randomized clinical trial investigating the safety and effectiveness of IA injection of a high-molecular-weight, linear chain hyaluronic acid produced by bacterial fermentation (IA-BioHA) vs. an avian-derived hyaluronic acid (hylan G-F 20), which uses cross-linking to achieve high molecular weight, for the treatment of symptomatic knee OA found significant differences favoring IA-BioHA in patient global assessment and percent of patients requiring rescue medication [13]. Moreover, this study reported a significantly higher incidence of post-injection effusions in the hylan G-F 20 group vs the IA-BioHA group. Consistent with the latter observation are reports that crosslinked, high molecular weight, avian-derived HA may elicit acute local reactions after intra-articular injection [8, 10, 23]. More recently, broad based analyses of clinical trials evaluating the effects of IA-HA in knee OA found higher molecular weight IA-HA products and those derived from bacterial fermentation are associated with superior efficacy and safety [1, 6].

IA-BioHA is indicated for the treatment of pain in OA of the knee in patients who have failed to respond adequately to conservative non-pharmacologic therapy and simple analgesics [25]. In a 26-week pivotal clinical trial, IA-BioHA treatment produced a significant reduction in pain (characterized using a visual analog scale) compared to patients treated with normal saline. Outside of the standard patient-reported outcomes, little is known regarding the histopathological changes that may underlie HA efficacy or formulation differences in clinical effects. To further investigate the potential benefits of IA-BioHA viscosupplementation on OA joint structure and to support future HA formulation development, we investigated the dose-related effects of IA-BioHA on knee joint cartilage, collagen, synovitis, inflammatory cytokine expression, and pain responses in a well-characterized medial meniscal tear (MMT) rat model of OA [11]. The joint lesions in the MMT rat model are morphologically similar to that seen in human OA but occur much more rapidly [4, 11]. In addition, as differences have been reported between the clinical performance of cross-linked vs. linear HA and avian- vs. fermentation-derived HA, we compared the effects of IA-BioHA with hylan G-F 20, a cross-linked, avian-derived HA, in the MMT model to investigate factors that might contribute to clinically observed differences.

## Methods

All experiments were conducted in accordance with the NIH Guide for the Care and Use of Laboratory Animals. The present study design and usage of 95 rats was approved by Bolder BioPATH’s Institutional Animal Care and Use Committee (Protocol BBP-008) and performed at Bolder BioPATH (Boulder, CO).

### Rat osteoarthritis model

Adult male Lewis rats (Charles River, Wilmington, MA), approximately 13 weeks old, were housed 2–5 per cage in OptiRat Plus multilevel cages (Animal Care Systems, Centennial, CO) and were allowed unrestricted access to rodent chow (Teklad, Envigo, Indianapolis, IN) and water. Rats were grouped prior to surgery to ensure the mean body weight of each group was similar. MMT surgery was performed as previously described [11]. Briefly, isoflurane-anesthetized rats had a skin incision over the medial aspect of the right knee to expose the medial collateral ligament (MCL), which was transected to expose the medial meniscus. A full-thickness cut was made in the meniscus at the approximate midpoint on the medial side, avoiding the area of the ossicles, to simulate a full- thickness tear. Skin and subcutis were closed with 4–0 Vicryl® (Ethicon, Somerville, NJ). Mean body weight after MMT surgery (study Day 0) was 321 +/− 14.5 g. As a control, a separate group of animals underwent a sham surgery in which the MCL was transected to open the joint, but the meniscus was left intact. On study Day 6, prior to the first treatment, incapacitance weight-bearing testing was performed (described in detail below), and the rats were randomized to maintain equal mean body weights as well as mean standing weight-bearing deficits across the groups.

### Study design and group allocation

Rats with MMT surgery were randomized into one of 5 IA injection treatment groups (*n* = 15 rats/group): 1 injection (Day 7 post-surgery) of 50 μl of vehicle (phosphate buffered saline, PBS), 1 injection of IA-BioHA (EUFLEXXA®, 10 mg/ml, Ferring Pharmaceuticals Inc., Parsippany, NJ), 1 injection of hylan G-F 20 (SynVisc One®, 8 mg/ml, Genzyme Corporation, Ridgefield, NJ); 3 injections (Days 7, 14, 21 post-surgery) of IA-BioHA, or 3 injections of vehicle. The sham operated rats (10 rats/group) were given an IA injection of PBS on Day 7. After treatment, all animals were evaluated at regular intervals for effects on body weight gain, knee swelling, standing, and moving pain using gait and incapacitance measures. On Day 35 post-surgery, joints were collected for microscopic evaluation of effects of treatment on joint morphology.

### Gait evaluation

For gait analysis, the difference in dynamic weightbearing between the ipsilateral (MMT operated; right side) limb and contralateral (control; left side) limb were determined as described previously [14].

### Incapacitance test

The incapacitance test quantifies the difference in hind paw weight distribution as an index of joint discomfort. The test was performed as previously described [5]. Incapacitance testing was performed on Day 6 (baseline), and Days 9, 16, 23, and 30. Prior to the first evaluation, the animals were trained at least twice on the procedure. At the time of testing, each animal was placed into a plexiglass housing of the incapacitance meter and allowed to acclimate. The difference in weight distribution between the right (OA model) and left (contralateral control) paws was determined. As a positive control group for the incapacitance test, 10 MMT animals with no IA injections received tramadol (50 mg/kg, oral) on the day of measurement (no microscopic evaluation was performed on tramadol-treated animals). The weight bearing differential (left-right) was plotted from Day 6 to Day 30 and the area under curve (AUC) was determined for each test group.

### Histological analysis

**Fig. 1:**
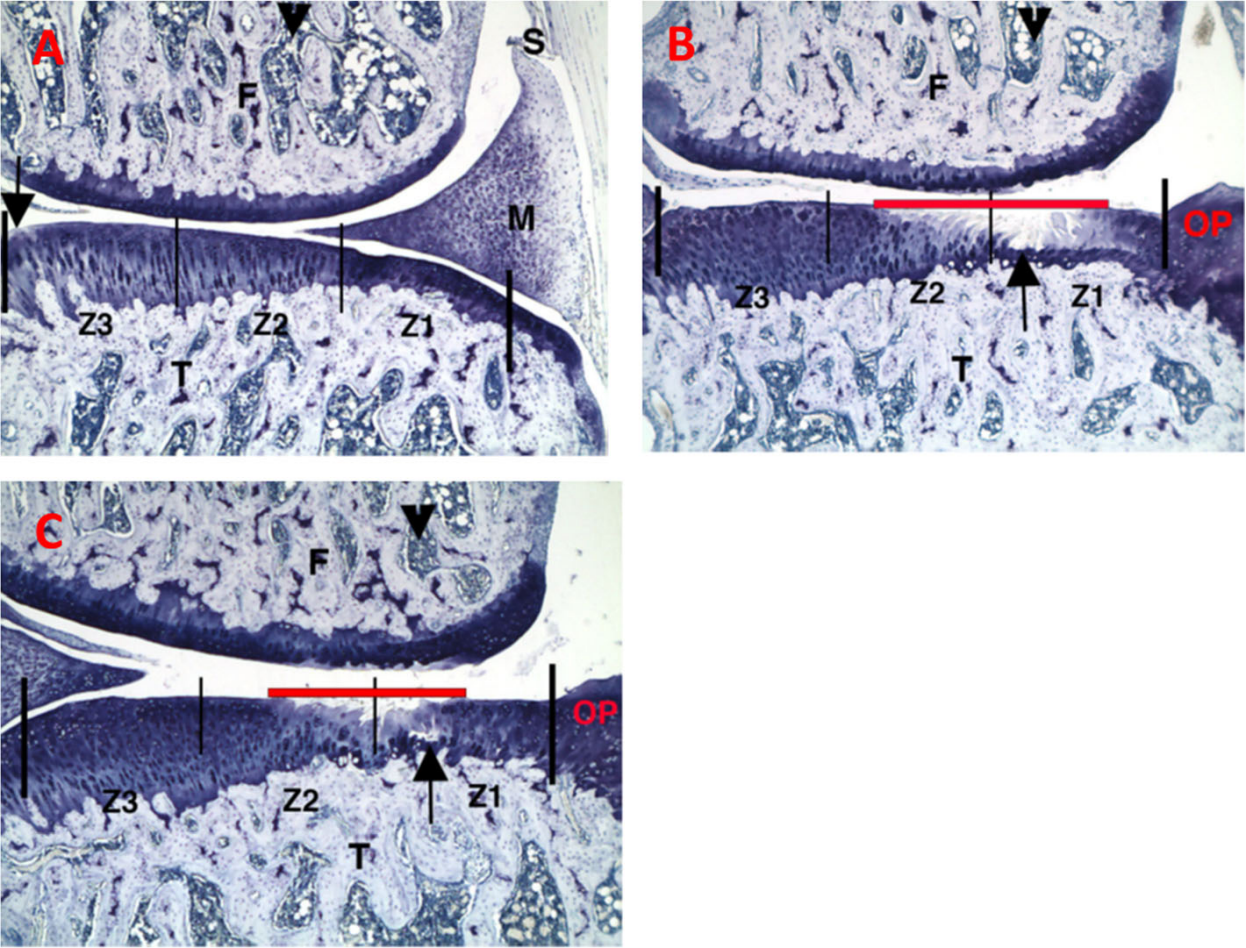
Representative histopathology photomicrographs stained with toluidine blue. **a** Sham control group, left knee, medial. **b** PBS injections at D7, D14, and D21, right knee, medial. **c** IA-BioHA injections (0.5 mg/rat) at D7, D14, D21, right knee, medial. Vertical bars delineate 2000 μm of tibial plateau and zones. F - femur; T - tibia; M - meniscus; S - synovium. Arrow identifies a small surface area of minor spontaneous cartilage degeneration in the area not protected by meniscus in zone 3. Arrowhead identifies bone marrow. Red horizontal line identifies extent of collagen degradation. Images acquired at 50x magnification

Microscopic analysis of joint sections was done using methods previously described [7] with minor modifications to fit the conditions of the present study. On Day 35, animals were anesthetized by CO_2_ inhalation and euthanized by bilateral pneumo-thoracotomy. The right knee of all animals was collected and placed in 10% neutral-buffered formalin and then decalcified in 10% formic acid for 3 days. Each joint was cut along the frontal plane into two halves and embedded in paraffin. Three sections were cut from each half in approximately 160-μm-thick sections, stained with toluidine blue, and analyzed microscopically. The following parameters were assessed by the pathologist blinded to the treatment group: Total Tibial Cartilage Degeneration Width; Substantial Tibial Cartilage Degeneration Width; Total Cartilage Degeneration Score, 3-zone Sum; Total Cartilage Degeneration Depth Ratio; Femoral Cartilage Degeneration Score Sum; Collagen Width – Total. Tibial cartilage degeneration was further scored for outer, middle and inner zones (see Fig. 1). Values for the parameters listed below were measured for the three sections and averaged for each animal (not performed for the tramadol positive control group).

### Immunohistochemistry (IHC)

Immunostaining for ED-1 (Bio-Rad, #MCA341), tumor necrosis factor alpha (TNFα) (Bio-Rad, #AAR33), and transforming growth factor beta (TGFβ) (Abcam Recombinant Antibodies, #ab170874) was performed in an exploratory manner on 2 joints per group that represented: 1) the approximate group mean, and 2) the joint with the most severe (i.e., worst) arthritis lesions in each group. One normal left joint from the vehicle control group was also evaluated.

Formalin-fixed, decalcified rat knees were trimmed and embedded in paraffin. Slides were then deparaffinized, rehydrated, exposed to antigen retrieval (heat-mediated or enzyme, if necessary), incubated with primary and secondary antibody. Slides were then counterstained with hematoxylin, dehydrated, and permanently cover-slipped.

ED-1 and TNFα scores were based on thickness and extent of infiltrate on medial and lateral aspects of the knee. TGFβ scoring was based on the distribution and intensity of staining vs. normal background staining.

### Data analysis

The sample sizes were determined based on representative data obtained from a previous study [11], which indicated that 15 rats per group would be enough to detect an approximately 25% difference in means for histopathologic parameters of interest with 80% power. Descriptive statistics (mean, standard error [SE]) were calculated for the assessments. Statistical analysis was performed on mean animal measures if the observations were taken in triplicate (immunocytochemistry, histology) or quadruplicate (gait). Treatment groups were compared to control groups for parametric data using a one-way analysis of variance (ANOVA) or a Student’s two-tailed t-test and for non-parametric data using a Kruskal-Wallis test with a Dunn’s post-hoc analysis or a Mann-Whitney U test. The significance level for all tests was *P* < 0.05. Data analysis was performed with Prism v7.0d (GraphPad), Minitab v18.1, and Microsoft Excel. We assumed that measured or continuous data were normally distributed, and, typically, the parametric tests are robust against violations of the normality assumption. We used non-parametric tests for data not assumed to be normally distributed, such as gait and pathology scores, and those tests do not make assumptions about the distribution.

## Results

### Body weight and gait

There were no notable changes in body weight in any of the study groups. The animals did exhibit mild changes in gait, consistent with what is expected after MMT surgery in the rat [14, 15].

### Histopathology

Representative histopathology sections for the sham control, vehicle (PBS)-injected and IA-BioHA-injected rats, 35 or 36 days after MMT, are shown (Fig. 1). Joints from rats undergoing sham surgery were mostly normal with occasional, very minimal, cartilage degeneration inside the medial tibia adjacent to the cruciate ligaments (zone 3, area not protected by the meniscus, see Fig. 1a). The joint pathology for vehicle injected rats was consistent with that reported previously for this model [11]. Briefly, minimal to severe collagen degeneration was seen on approximately half of the tibial surface of most control joints. Tibial cartilage damage was most severe on the outside of the medial tibia (zone 1; Fig. 1b) and was very minimal on the inner 1/3 (zone 3; Fig. 1). Large osteophytes and very minimal to mild synovitis were observed in all vehicle control and treated animals.

**Fig. 2:**
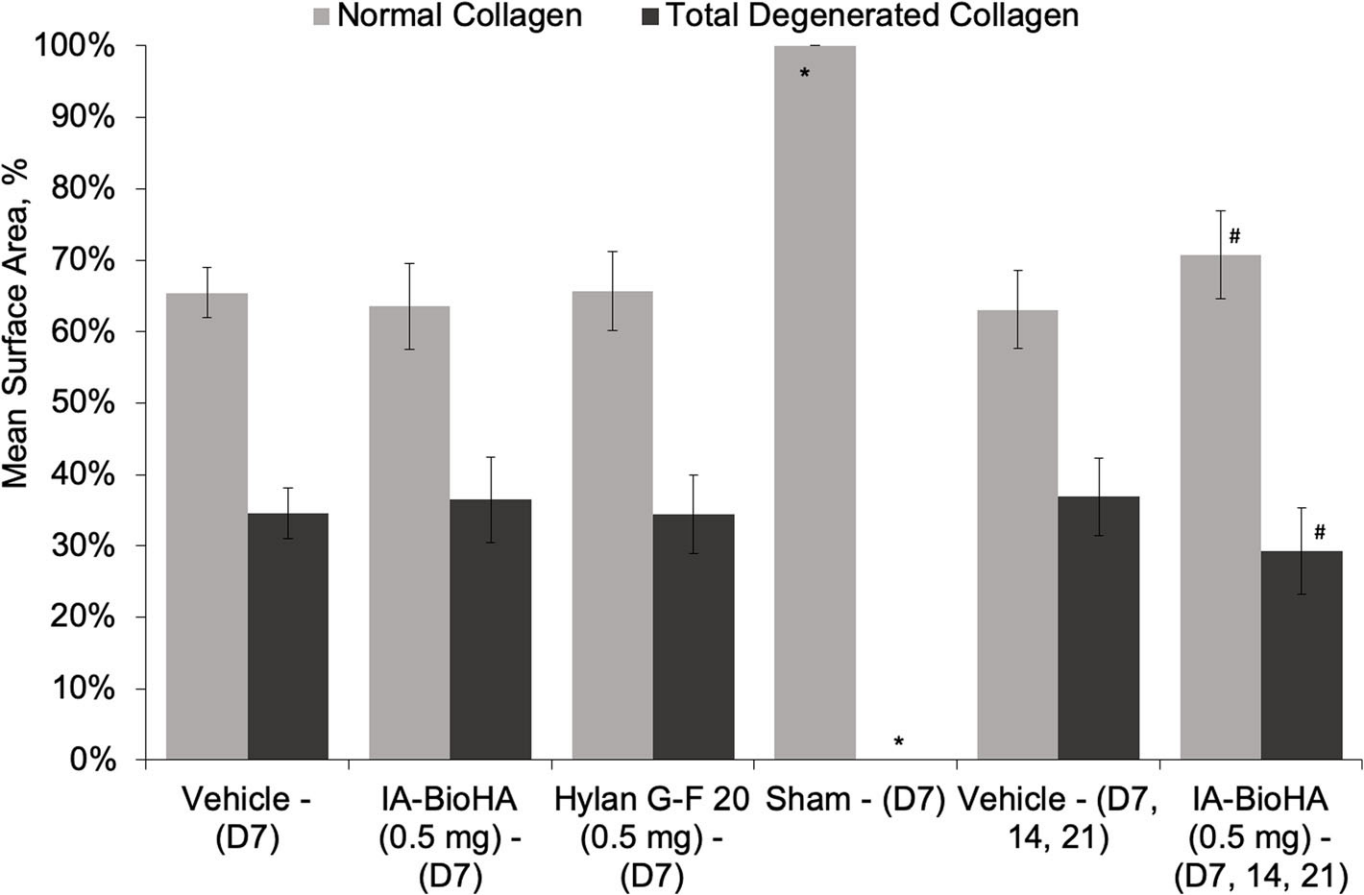
Histological scoring for tibial collagen degeneration and amount of normal collagen. Collagen damage is measured with an ocular micrometer as the width of any collagen damage across the tibial plateau. The mean proportion of the tibial surface that showed either normal collagen or total collagen degeneration is shown. Three weekly injections of IA-BioHA showed a significantly greater amount of normal collagen and significantly smaller amount of total collagen degeneration compared to vehicle control. Error bars indicate 1 standard error from the mean. *n* = 15/group for test article and vehicle control groups, *n* = 10/group for sham surgery. * different from the D7 and D7, 14, 21 vehicle control groups, *p* < 0.0001. ^#^ different from the respective D7 or D7, 14, 21 vehicle control *p* = 0.011

Rats dosed on Days 7, 14, and 21 with IA-BioHA had significantly reduced total and mild collagen degeneration as compared to vehicle control rats (see Fig. 1c). Morphometric measurement of tibial collagen width indicated three weekly IA injections of IA-BioHA significantly reduced collagen degeneration and increased normal collagen by 21% vs. the respective vehicle group (Fig. 2, Table 1). No significant change in collagen degeneration was observed for rats given a single IA injection of hylan G-F 20 or IA-BioHA compared to vehicle-injected rats.

**Table 1:** Summary of Knee Morphometry Parameters

	Single Injection^**1**^					Three Injections^**1**^		
	Vehicle (PBS) Control	IA-BioHA		Hylan G-F 20		Vehicle (PBS) Control	IA-BioHA	
Mean (SE)			% Change^2^		% Change^2^			% Change^2^
Total tibial cartilage degeneration width (μm)	1926.7 (17.9)^2^	1797.8 (54.0)	**−7.6**	1853.3 (50.3)	**−4.3**	1897.8 (22.4)	1780. 0 (59.2)	**−7.0**
Substantial tibial cartilage degeneration width (μm)	653.3 (69.3)	624.4 (63.3)	**−4.4**	693.3 (65.1)	6.1	620.0 (69.9)	573.3 (52.5)	**−7.5**
Total cartilage degeneration score, 3-zone sum	6.0 (0.5)	5.73 (0.4)	**−5.1**	6.3 (0.5)	4.4	6.2 (0.5)	5.3 (0.4)	**−15.4**
Total cartilage degeneration depth ratio, mean (μm)	0.33 (0.03)	0.32 (0.03)	**−2.6**	0.35 (0.03)	4.6	0.34 (0.03)	0.29 (0.03)	**−14.6**
Femoral cartilage degeneration score	2.3 (0.35)	1.8 (0.24)	**−20.2**	2.8 (0.33)	24.1	1.8 (0.28)	1.3 (0.25)	**−24.4**
Collagen width – total (μm)	0.35 (0.02)	0.36 (0.03)	**−5.6**	0.34 (0.02)	0.5	0.37 (0.02)	0.29 (0.02)	**−20.7**

*Abbreviations*: *IA-BioHA* Intra-articular biologically derived hyaluronic acid, *PBS* Phosphate buffered saline

^1^*N* = 15 animals/group. Results represent the mean (SE)

^2^Analysis of the percent change relative to vehicle control across all histopathology parameters demonstrated a statistically significant difference in the number of improved responses after a single (*p* < 0.0001) or three (*p* < 0.0001) weekly injections of IA-BioHA; the difference between vehicle control and a single injection of hylan G-F 20 was not significant

Morphometric measurement of joint cartilage histopathology for rats receiving a single IA injection of hylan G-F 20, single injection of IA-BioHA or 3 weekly injections of IA-BioHA relative to those for vehicle control-treated rats, are summarized in Table 1. Analysis of the percent change from vehicle control across all histopathology parameters demonstrated a statistically significant difference after 1 (*p* < 0.0001) or 3 (*p* < 0.0001) weekly injections of IA-BioHA compared to a single injection of hylan G-F 20.

**Fig. 3:**
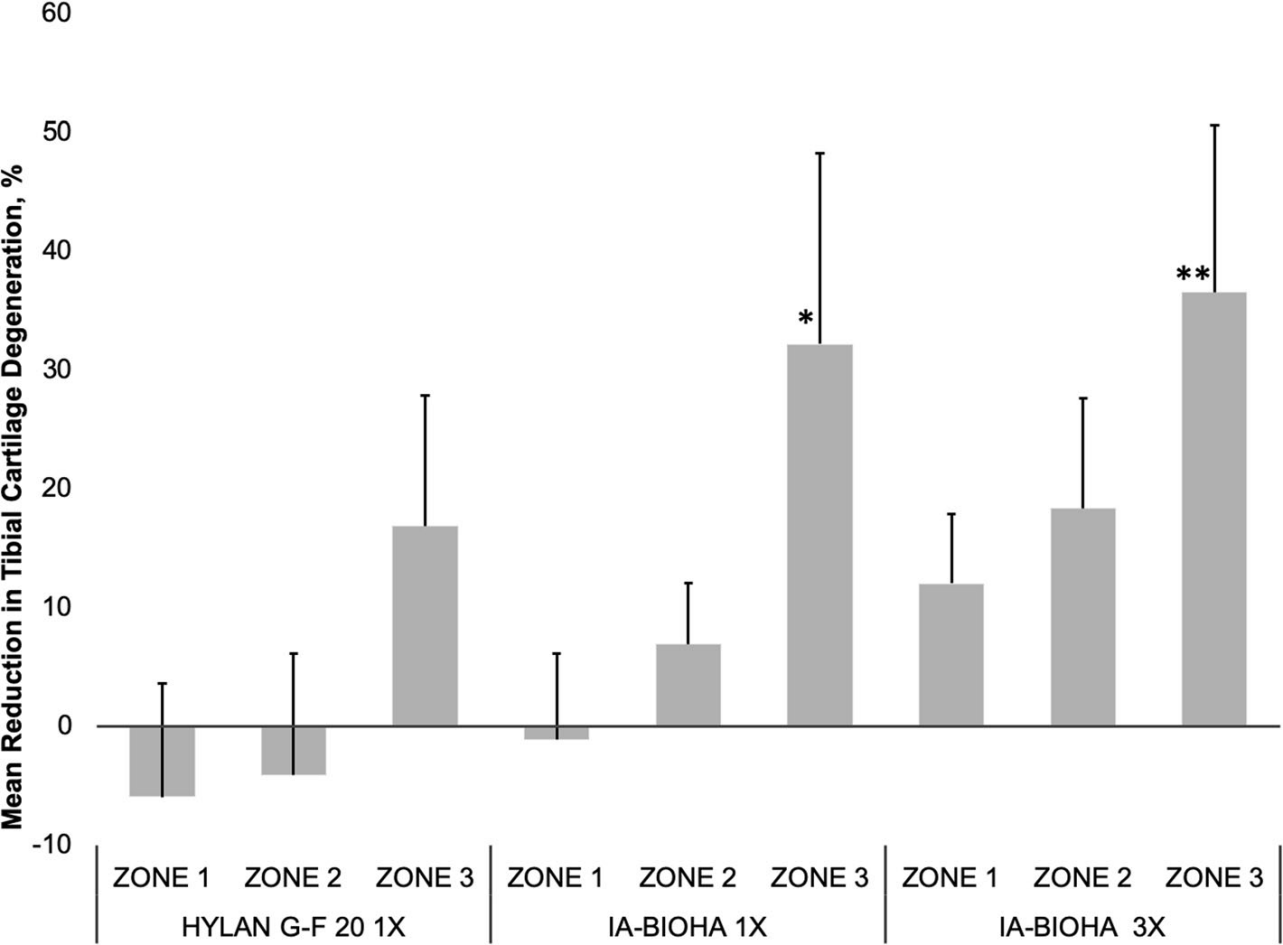
Medial tibial collagen degeneration by tibial zone. The effect of IA-BioHA or IA-hylan G-F 20 on tibial collagen by tibial zone is presented as the percent change from the vehicle control animals. A positive number indicates the tibial collagen score was less than vehicle control and a negative number indicates the tibial collagen was greater than vehicle control. Error bars reflect 1 standard deviation. *n* = 15/group. Different from vehicle control; * *p* = 0.016, ** *p* = 0.030

As previously described for this OA model [4], cartilage degeneration is most severe in the outer medial tibia zone (Fig. 1, zone 1) with decreasing degeneration toward the innermost zone (Fig. 1, zone 3). There were differences in the effectiveness of HA treatment by tibial zone, type of HA, and number of HA injections, as assessed by reduction in cartilage degeneration relative to vehicle-injected animals (Fig. 3). One or 3 injections of IA-BioHA significantly improved cartilage degradation scores at tibial zone 3 (*p* < 0.011 vs vehicle control; *n* = 15) but not for zones 1 and 2, which had more severe cartilage degeneration. A statistically significant difference in the reduction of cartilage degeneration relative to vehicle control was not detected for animals treated with hylan G-F 20 for any zone, although a trend in the results suggest some benefit in zone 3.

### Immunocytochemistry

**Fig. 4:**
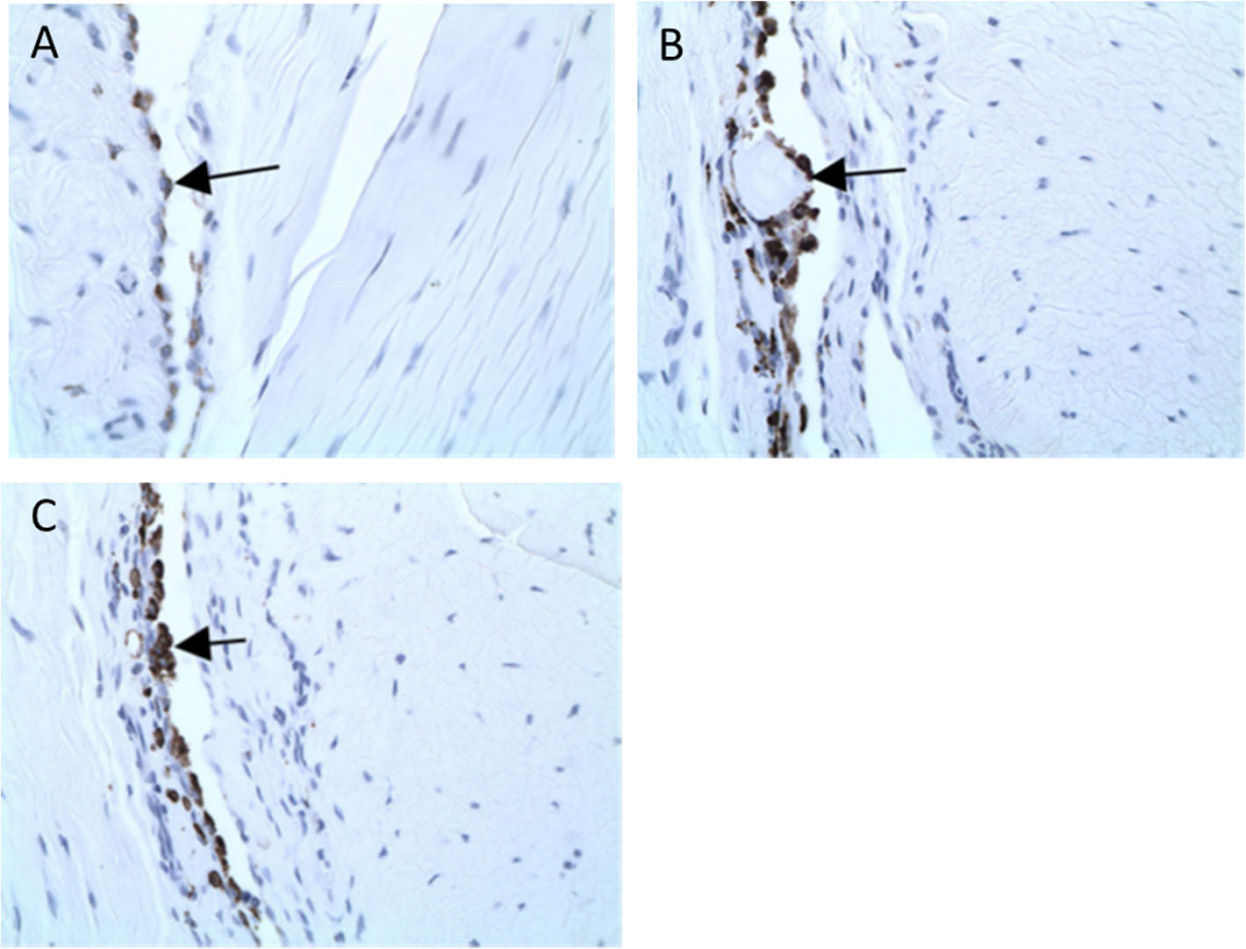
ED-1 staining on the lateral tibial surface. **a** Uninjected left knee. Arrow identifies normal background level of ED-1-positive macrophages in synovium. **b** PBS injections at Days 7, 14, and 21 in the right knee. Arrow identifies ED-1-positive macrophages surrounding degenerating cartilage fragment in synovium. There is a mild increase over normal background levels, likely as a result of the disease process (rather than the treatments). **c** IA-BioHA injections (0.5 mg/rat) at Days 7, 14, and 21 in the right knee. Arrow identifies ED-1-positive macrophages in synovium. There is a mild increase over normal background levels, likely as a result of the disease process. Images were acquired at 400x magnification

**Fig. 5:**
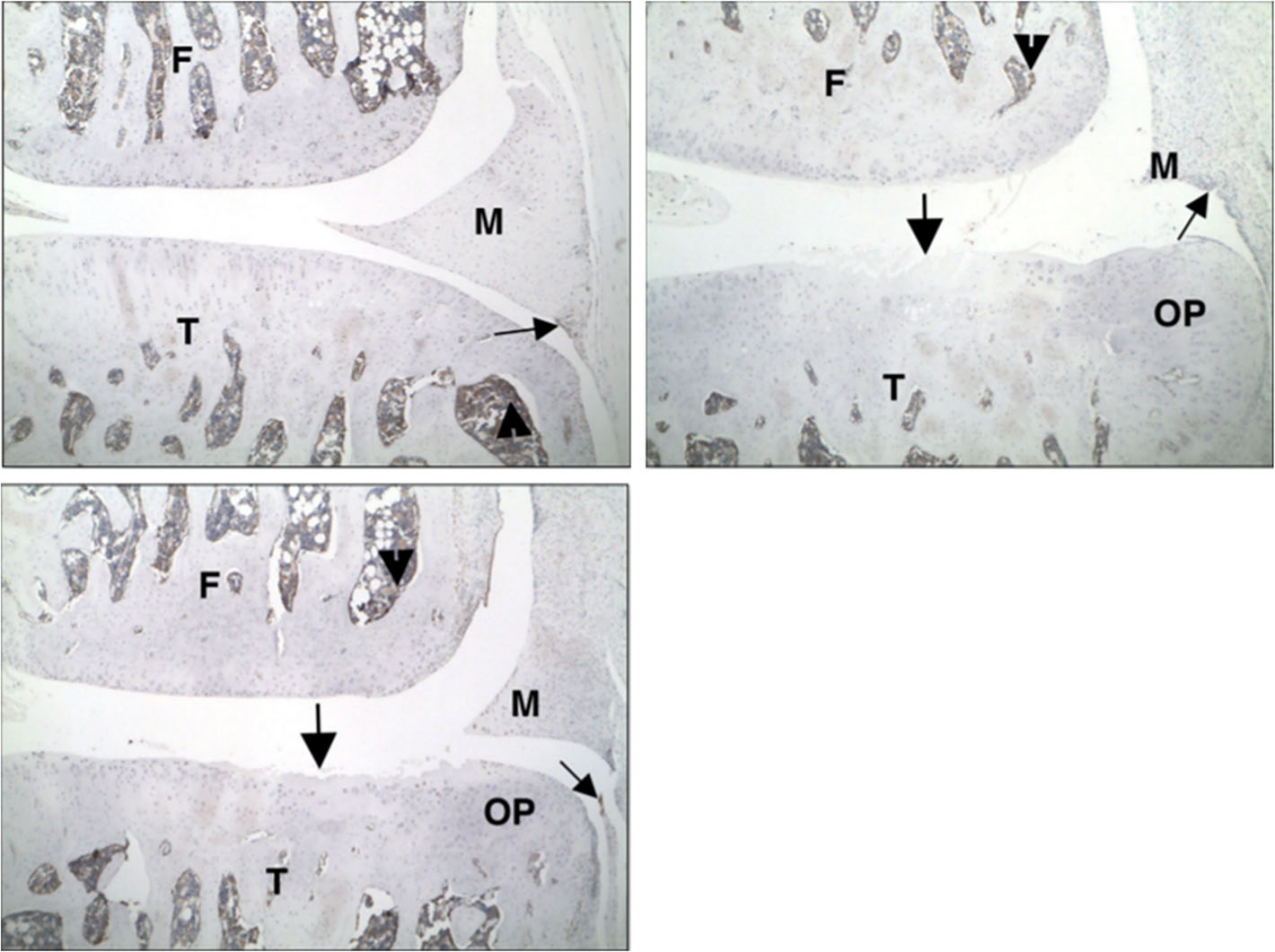
TNFα staining on the medial tibial surface. **a** Uninjected left knee. **b** PBS injected in the right knee. **c** IA-BioHA (0.5 mg/rat) injected at Days 7, 14, and 21 in the right knee. The large arrow identifies a cartilage lesion. There are numerous TNFα-positive cells in the bone marrow (arrowhead) and minimal multifocal-positive cells in the synovium (small arrow). The disease process and IA-BioHA treatment did not result in a significant increase in TNFα immunostaining. F – femur; M – meniscus; OP – osteophyte; T – tibia. Images were acquired at 50x magnification

**Fig. 6:**
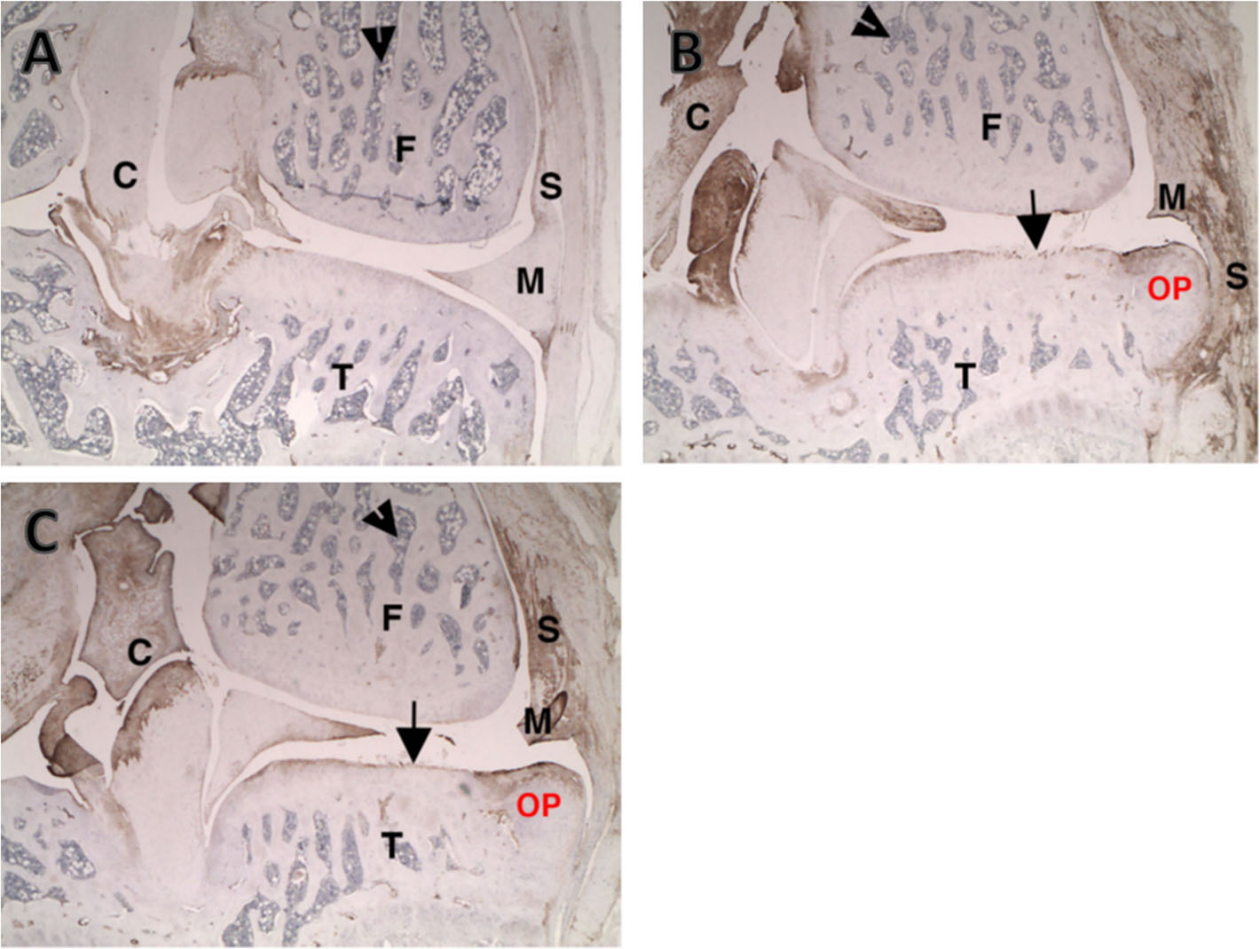
TGFβ staining on the medial tibial surface. **a** Uninjected left knee. Arrowhead identifies bone marrow. Note normal immunopositivity in connective tissue of synovium (S), collateral ligament joint capsule, and cruciates (small C). **b** PBS injected right knee. Note increased connective tissue staining in healing medial synovium (S) damaged meniscus (M), cruciates (small C), and osteophyte (OP). The damaged tibial cartilage has increased staining of the tangential layer in areas where it is intact. **c** IA-BioHA (0.5 mg/rat) injected at Days 7, 14, and 21 in the right knee. Note increased connective tissue staining in healing medial synovium (S) damaged meniscus (M), cruciates (small C), and osteophyte (OP). The damaged tibial cartilage has increased staining of the tangential layer in areas where it is intact. F- femur; M – meniscus; T – tibia. Images were acquired at 25x magnification

The degree of synovitis and inflammatory cytokine expression was evaluated by immunocytochemistry using antibody to the macrophage-specific antigen: ED-1 (Fig. 4), TNFα (Fig. 5), and TGFβ (Fig. 6). The normal left knee exhibited only background staining. ED-1 immunostaining in the right knee of vehicle control-treated rats was very minimally increased compared to the left knee and was associated with macrophages in the synovium and focal increases in osteoclasts in areas of bone damage subjacent to cartilage lesions and in osteophytes.

TNFα immunostaining was minimally increased and associated predominantly with synovial macrophages. Immunostaining of rats given a single injection of hylan G-F 20 or 1 or 3 weekly injections of IA-BioHA was similar to vehicle control-treated rats, with very minimal or minimal ED-1 staining and TNFα staining. Vehicle control-treated rats had a moderate increase in TGFβ staining in all normally stained areas, articular cartilage, and osteophytes. Rats injected with hylan G-F 20 or 1 or 3 weekly injections of IA-BioHA were similar to vehicle control-treated rats with moderate TGFβ staining.

### Pain behavior

**Fig. 7:**
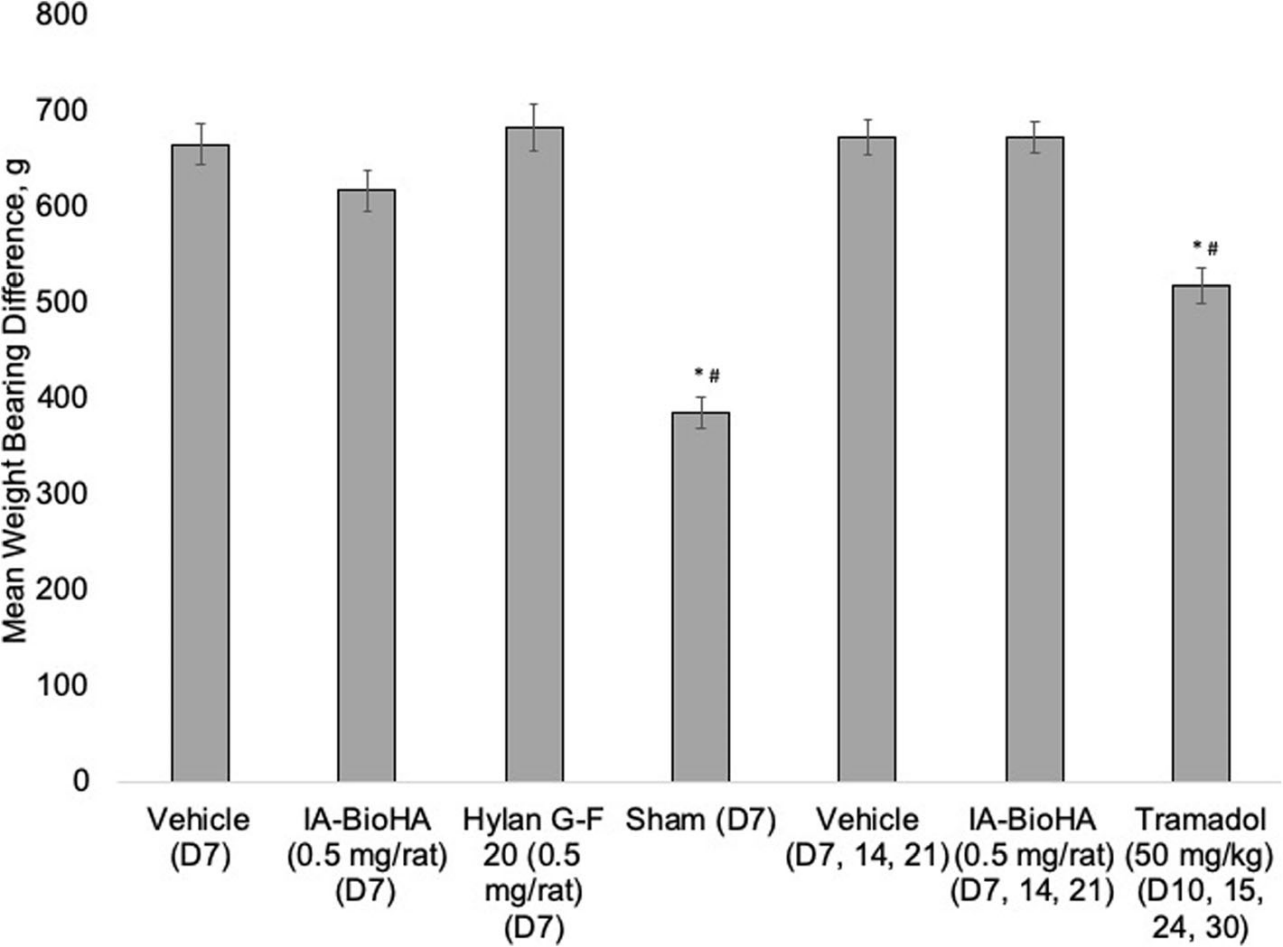
Incapacitance testing: weight bearing differential (left-right). Results shown are area under the curve. Error bars represent 1 standard error from the mean. *n* = 15 rats/group for test article and vehicle control, *n* = 10 rats/group for sham surgery and tramadol groups. * *p* = 0.001 Student’s *t*-test vs. vehicle (PBS) D7. ^#^*p* = 0.001 Student’s *t*-test vs. vehicle (PBS) D7, 14, 21

Treatment with tramadol, the analgesic control, on the day of incapacitance testing significantly improved test performance (Fig. 7). Injection of vehicle or any of the HA treatment regimens did not have a detectable effect on incapacitance test results.

## Discussion

MMT surgery of rats is a well-characterized preclinical model of knee osteoarthritis, which produces a gradient of injury across the tibial surface [7, 11]. The cartilage changes in this model are similar to those observed in humans with osteoarthritis [3, 7, 11]. Moreover, the rapid progression of the cartilage degeneration after MMT surgery provides a high hurdle for detecting efficacy of therapeutic interventions. Results from the present study extend our understanding of the potential benefits of IA-BioHA on the knee joint by demonstrating a single IA injection or 3 weekly injections of 50 μg IA-BioHA initiated 1 week after surgery were effective in reducing tibial cartilage and collagen damage in a rat model of knee osteoarthritis. IA-BioHA is a non-crosslinked, linear chain, high-molecular-weight (2.4–3.6 million Da) product produced by bacterial fermentation indicated for the treatment of pain in OA of the knee in patients who have failed to respond adequately to non-pharmacologic therapy. These findings support the reasoning that viscosupplementation of IA-BioHA in patients with knee osteoarthritis has the potential to delay disease progression.

As illustrated in Fig. 1, the most severe damage occurs in the outer region of the tibia, with less injury toward the inner tibial surface. This gradation of response enables an evaluation of IA HA benefits under varying degrees of osteoarthritic injury. The present study demonstrated the beneficial effects of HA on tibial cartilage differed by type of HA, HA dose, and tibial surface zone. The collagen-sparing activity of IA-BioHA was greatest after 3 weekly injections of IA-BioHA, and modest by single injection of IA-BioHA, with the most pronounced effect on the inner tibia. These results suggest that IA-BioHA treatment may be most effective when initiated early in the progression of OA when the joint injury is less severe.

The present study also assessed the effects of HA treatment on pain using the incapacitance test, which measures changes in hind paw weight distribution as an index of joint discomfort [5]. In agreement with previous studies using rodent OA models, significant changes in weight bearing were observed with the MMT model. While administration of the opioid tramadol significantly reduced joint discomfort, no significant effect was detected for any of the HA treatments. The absence of an apparent effect of IA-BioHA on the pain response may related to the nature of the MMT model, which produces a graded injury across the tibial surface. It is possible the severe joint damage occurring on the outer tibial region, which was least affected by HA viscosupplementation, produces enough discomfort that any benefit in other zones from HA injection cannot be detected by the incapacitance test. The observation that the opioid tramadol produces only a 25% improvement in weight distribution further supports that the incapacitance test may have limited sensitivity for treatments that moderately effect pain.

The results of the present study suggest differences between IA-BioHA and hylan G-F 20 in their ability to spare OA joint connective tissue. Collectively, the beneficial effects of 1 or 3 IA-BioHA injections were significantly greater than those seen with injection of hylan G-F 20, which did not produce a detectable effect on joint cartilage or collagen in the MMT rat OA model. Moreover, as illustrated in the tibial zone analysis, there were clear differences in the degree of cartilage sparing between single injections of IA-BioHA and hylan G-F 20. Consistent with the present findings for hylan G-F 20, Yanagisawa et al. reported that a single IA injection of 50 μg of hylan G-F 20 in partially-meniscectomized rats did not reduce histologically-examined joint cartilage damage, but found that 4 or 8 weekly injections of hylan G-F 20 were effective in preserving cartilage [24]. These results, combined with our findings, suggest that repetitive doses of hylan G-F 20 are required to reduce joint injury surgical models of rat OA. Like the observations in the MMT model, clinical data suggest differences in responses of patients with OA to IA-BioHA and hylan G-F 20. For example, a prospective, multicenter, randomized, double-blind clinical trial on patients with knee OA given 3 weekly IA injections of BioHA or hylan G-F 20 found similar effects on the Western Ontario and McMaster Universities Osteoarthritis Index (WOMAC) pain score, but a greater benefit of IA-BioHA vs. hylan G-F 20 in patient global satisfaction and reduction in the percentage of patients requiring rescue medication [13]. We speculate that the greater collagen sparing activity observed in the MMT rat model for IA-BioHA may contribute to improved outcomes seen in patients with OA.

In the present study, synovitis (characterized by histopathology); and immunostaining for ED-1 (macrophage antigen), for the inflammatory cytokine TNFα, and for TGFβ, were minimal and not affected by IA injection of IA-BioHA or hylan G-F 20. Some studies have suggested that HA can directly regulate inflammatory and immunoregulatory processes [19]. The absence of any effects of IA-BioHA on macrophage recruitment and inflammatory cytokine expression, while reducing cartilage and collagen damage, implies that IA-BioHA did not have a direct effect on the inflammatory process, and the beneficial effects on connective tissue were like due to its viscoelastic properties providing cushioning and lubrication.

Whether differences in outcomes for animals treated with IA-BioHA or hylan G-F 20 are due to the nature of the devices; IA-BioHA being non-crosslinked and fermentation derived; or some other factors, was not addressed in the present study. In a study of a low molecular weight (800 kDa), non-crosslinked, avian derived HA (Artz, Seikagaku Corp., Tokyo, Japan), Jean et al. found that 5 weekly IA injections of 100 μg HA reduced cartilage damage and synovitis in rats after anterior cruciate ligament transection (ACLT model) [12]. The ACLT model is a slow-developing model of OA, requiring 8–12 weeks for substantial joint damage (vs 2–3 weeks for the rat MMT model) and, the dose used by Jean et al. was twice that used in the present studies (100 vs 50 μg per IA injection). However, the results for Artz-treated animals suggest that, regardless of molecular weight and source, HA can positively affect OA joint cartilage. It will require further head-to-head testing of HA devices to determine whether size, source, or crosslinking is a factor influencing differences seen in connective tissue preservation for joints treated with IA-BioHA or hylan G-F 20.

There are some limitations in the present study. First, while the rat MMT model produces lesions that are morphologically similar to human disease, cartilage degeneration progresses rapidly, presenting an obstacle for detecting protective effects of devices and drugs. In the present study, while some results suggested BioHA had a greater beneficial effect on joint cartilage than a single dose of hylan G-F 20, a detailed comparison of the dose-related and temporal pattern of response would be needed to draw firm conclusions on differences. Additionally, we did not detect an effect of IA hyaluronic acid on inflammatory cytokine expression. However, our histological evaluation was only exploratory in nature, examining a single time point 35 days after MMT surgery and 28 days after initiating HA treatment. It is possible that changes in cytokine expression occur at an earlier point in disease progression.

## Conclusion

The present studies demonstrate the ability of IA-BioHA viscosupplementation to preserve joint cartilage and collagen in a well-established rat model of knee OA. Moreover, the observation that IA-BioHA had its greatest effect on tibial surfaces with less severe injury suggesting that IA-BioHA treatment should be initiated as early as possible when the joint injury is less severe to have the greatest effect on progression of osteoarthritis disease. A comparison of the responses to IA-BioHA with those of hylan G-F 20 in the MMT rat OA model suggest IA-BioHA may be more effective in preserving joint connective tissue, an observation consistent with other preclinical and clinical studies indicating differences amongst HA device efficacy.

## Data Availability

The datasets used and/or analyzed during the current study are available from the corresponding author on reasonable request.

## Abbreviations

ACLT: Anterior cruciate ligament-transection
ANOVA: Analysis of variance
AUC: Area under the curve
HA: Hyaluronic acid
IA: Intra-articular
IA-BioHA: Intra-articular injection of biologically derived hyaluronic acid
MCL: Medial collateral ligament
MMT: Medial meniscal tear
OA: Osteoarthritis
PBS: Phosphate buffered saline
SE: Standard error
TGF: Transforming growth factor
TNF: Tumor necrosis factor
